# Effects of couple-based violence prevention education on male partners' knowledge, attitudes and controlling behavior related to intimate partner violence in rural Ethiopia: a cluster randomized controlled trial

**DOI:** 10.3389/fpubh.2025.1506459

**Published:** 2025-03-10

**Authors:** Zeleke Dutamo Agde, Jeanette H. Magnus, Nega Assefa, Muluemebet Abera Wordofa

**Affiliations:** ^1^Department of Population Study and Family Health, Institute of Health, Jimma University, Jimma, Ethiopia; ^2^Department of Reproductive Health, College of Medicine and Health Sciences, Wachemo University, Hossana, Ethiopia; ^3^Faculty of Medicine, University of Oslo, Oslo, Norway; ^4^College of Health and Medical Sciences, Haramaya University, Harar, Ethiopia

**Keywords:** attitude, knowledge, intimate partner violence, male partners, rural Ethiopia

## Abstract

**Background:**

Supportive attitudes toward wife-beating and the experience of controlling behavior from husbands have been known to increase the risks of intimate partner violence (IPV). The aim of this study was to determine the effects of couple-based violence prevention education in addressing IPV-related knowledge, attitudes, and controlling behavior among male partners in rural Ethiopia.

**Method:**

A cluster randomized controlled trial was conducted using a two-arm parallel group design. The 16 clusters were randomly allocated into 8 intervention groups and 8 control groups. A total of 432 couples (432 male partners and 432 pregnant wives) participated in the trial. Couple-based violence prevention education (CBVPE) was provided to the participants in the intervention group, while the control group received routine or standard care. Difference-in-difference analysis and the Generalized Estimating Equation (GEE) model were used to assess the effectiveness of the intervention.

**Result:**

At the endline, 94.4% of male partners in the intervention group and 94.9% in the control group were available for the intention-to-treat analysis. Male partners in the intervention group were 3.7 times more likely to have good knowledge about IPV compared to male partners in the control group (AOR = 3.7; 95% CI 2.6–5.4). Male partners in the intervention group were 67.6% less likely to report supportive attitudes toward wife–beating compared to those in the control group (AOR = 0.324; 95% CI 0.229–0.459). Also, the proportion of controlling behavior exhibited by male partners in the intervention group was 56.4% less compared to the control group (AOR = 0.436; 95% CI 0.317–0.600).

**Conclusion:**

The intervention proved effective in enhancing knowledge about IPV, reducing supportive attitudes toward wife-beating, and curbing controlling behaviors among male partners in the study setting. This approach holds promise for scaling up and adapting to similar contexts in Ethiopia.

**Trial registration:**

The trial was registered on ClinicalTrials.gov with the identifier NCT05856214 on May 4, 2023.

## Introduction

Intimate partner violence (IPV) remains one of the major challenges in many countries in the world, especially Sub-Saharan Africa, including Ethiopia (1). Globally, one in three women has experienced physical or sexual violence by an intimate partner (2). Intimate partner violence not only violates human rights, but it also raises serious public health issues (3, 4). It is linked with significant health (5–9), social (10), and economic risks (11–13). In many African societies, including Ethiopia, men use violence as a means of inducing fear, control, and dominance over women (14).

Studies have shown that a considerable proportion of women in Ethiopia, ranging from 26% to 65%, experience some form of IPV during pregnancy (15–20). Furthermore, a systematic review and meta-analysis conducted in Ethiopia has revealed that the prevalence of IPV during pregnancy is 26.1% (21).

Violence results from the complex interplay of individual, relationship, community, and societal factors (22, 23). Research has shown that younger age of women, low educational status, being a housewife, jealousy, financial constraints, provocations by women, rigidity in gender roles, partner alcohol use, women's lower autonomy in household decision-making, deficiency in socio-emotional skills, gender inequality and living in a rural setting are contributing factors to IPV (16, 19–21, 24–27).

One of the significant risk factors for IPV is the attitude toward wife beating held by male perpetrators, as evidenced by the available body of literature (28–30). These attitudes are often shaped by deep-rooted cultural norms and societal expectations that view the subordination of women as acceptable or even necessary for maintaining social order (31, 32). According to the Ethiopian Demographic and Health Survey (EDHS) 2016 report, a considerable proportion of men (27.7%) and women (63.0%) believe that under certain circumstances, such as neglecting children, arguing with the husband, going out without telling the husband, burning food, and refusing to have sexual intercourse with the husband, wife-beating is justified (33). Controlling behavior includes a variety of actions used to create a sense of power and dependency, such as cutting someone off from their support networks, taking advantage of their skills and resources for one's own gain, denying them the ability to be self-sufficient, and controlling their day-to-day activities (34). Controlling behavior exhibited by husbands against their wives in at least one circumstance is 57.0%, according to the EDHS 2016 report (33).

Moreover, gender socialization reinforces gender inequalities and influences the behavior of men by reinforcing male dominance and female submissiveness (35). The hegemonic masculinity proposed by (36) provides a theoretical framework to understand how the dominant ideals of manhood centered on power, control, and emotional stoicism contribute to IPV perpetrations. Men adhering to the masculinity norms may use violence as a means to assert authority or respond to perceived threats to masculinity (36). Empirical studies have demonstrated that men endorsing traditional masculinity norms are more likely to justify IPV and engage in controlling behaviors (37, 38).

Poor knowledge about IPV, supportive attitudes toward wife-beating, and experiencing controlling behavior from the husbands are known to increase the risks of IPV (28, 29, 34, 39–43). Studies have found that involving men in efforts to prevent IPV is one of the effective strategies (44, 45). Couple-based interventions that raise awareness about IPV during pregnancy and its consequences, address effective communication and conflict resolution skills, gender norms, and power dynamics have demonstrated effectiveness in reducing IPV (46, 47). However, there is limited evidence on interventions that effectively address IPV-related knowledge, attitudes, and controlling behavior exhibited by husbands toward their wives in Ethiopia.

The trial's findings help in achieving Sustainable Development Goal (SDG) 5.2—eliminating all kinds of violence against women and girls by 2030 (48). It also offers insightful information to policymakers, emphasizing the significance of including couple-based violence prevention in Ethiopia's Health Extension Program (HEP) and the Health Sector Development Plan (HSDP). In this study, we examined the effectiveness of couple-based violence prevention education in improving IPV-related knowledge, attitudes, and controlling behavior among male partners in rural Ethiopia.

## Methods

### Study design, setting, period, and participants

The study design was a cluster-randomized controlled trial with two parallel arms. The study was conducted in rural districts of Hadiya Zone, Central Ethiopia. Additional details about the study setting can be found in our published study protocol (49). The recruitment and enrollment of the participants, including baseline assessment, were conducted in June and July, 2023. The intervention was implemented from August 1, 2023, to February 30, 2024. The endline assessment was conducted after 2 months of the completion of the intervention in April 2024. The trial included couples (male partners and their pregnant wives).

### Eligibility of participants, clusters, and health extension workers

The Health Extension Workers (HEWs) logbook review and pre-survey were performed in July 2023 to identify eligible couples. The trial included male partners living with their wives and couples who had at least one live childbirth. The couples, or either of them planned to move out of the study setting during the intervention implementation period, were excluded from the study.

This study considered kebeles, the lowest administrative units in Ethiopia, as clusters. To minimize information contamination between the control and intervention clusters, we implemented separation and buffer zone techniques. This involved selecting non-adjacent kebeles for the intervention and control groups. Health extension workers from the selected kebeles were chosen as implementers of the intervention. Their ability to speak the local language (Hadiyisa) was also used as an inclusion criterion for their selection as implementers of the intervention.

### Sample size determination

The trial's sample size was determined using Stata version 16.0, taking into account the following assumptions: an effect size of 20%, an IPV prevalence of 37.5% (22), an intra-cluster correlation coefficient (ρ) of 0.05 (44), a power of 80%, and an alpha of 5% for a two-tailed test. To account for the lack of independence within clusters and improve study power, a design effect of 2.3 was applied using the formula 1+ρ (m-1), where ρ is the intra-cluster correlation coefficient and m is the cluster size of 27. Considering a 20% loss to follow-up, the trial included a total of 432 couples (432 pregnant women and 432 male partners/husbands).

### Sampling procedure

**Figure 1:**
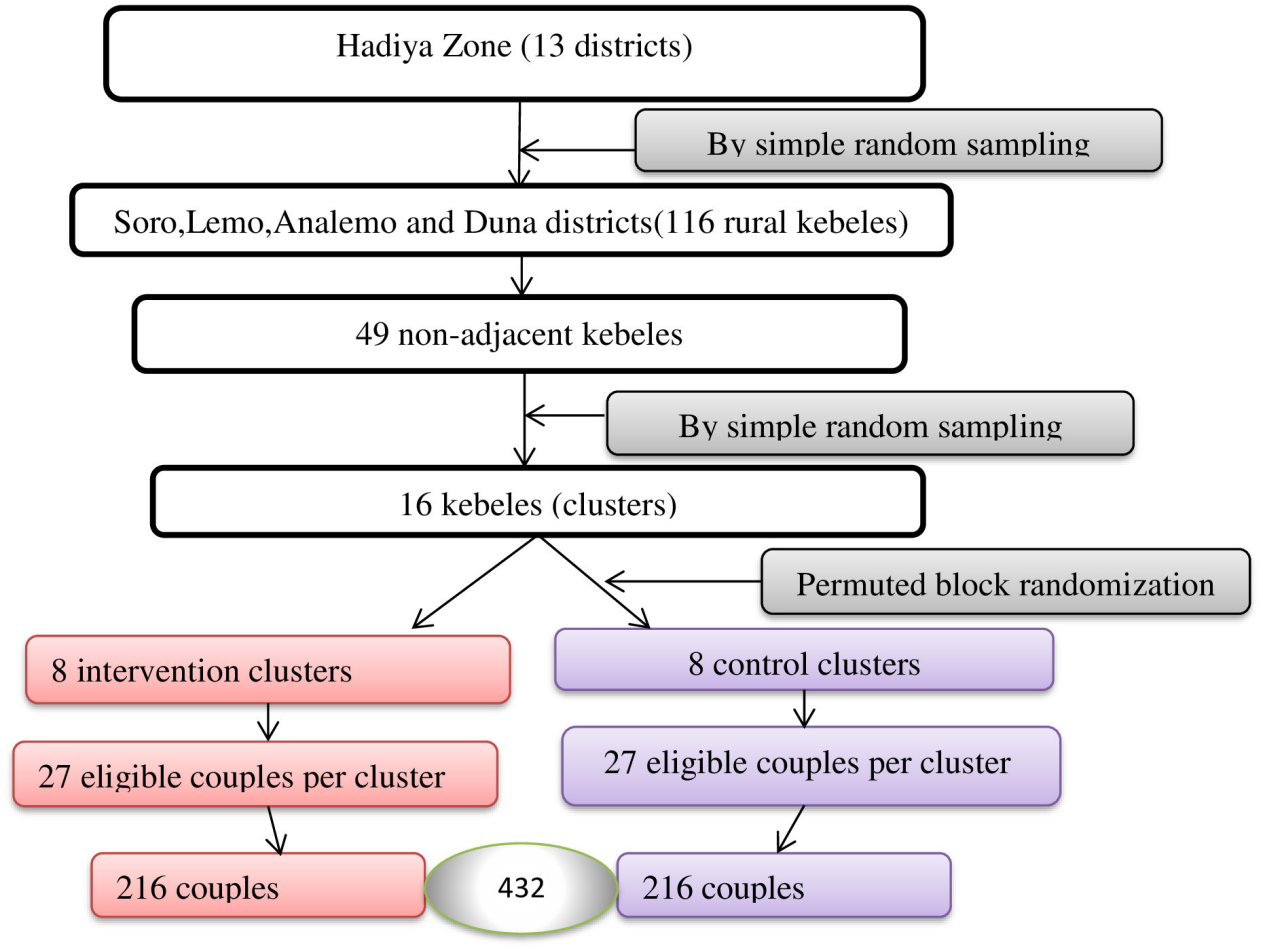
Schematic representation of sampling procedures for study participants in the rural districts of Hadiya Zone, Central Ethiopia, 2023/2024.

Four rural districts, namely, Soro, Lemo, Analemo, and Duna, were selected out of 13 districts in Hadiya Zone. We identified 49 non-adjacent kebeles from a total of 116 rural kebeles in these districts. Then, out of the 49 kebeles, 16 kebeles were selected through simple random sampling. These 16 kebeles were then randomly assigned into two groups-−8 intervention clusters and 8 control clusters. Within each of the 16 clusters, 27 eligible couples were selected, resulting in a total of 216 couples in both the intervention and control groups, and a total of 432 couples were included in the study (Figure 1).

### Randomization of clusters and blinding

**Figure 2:**
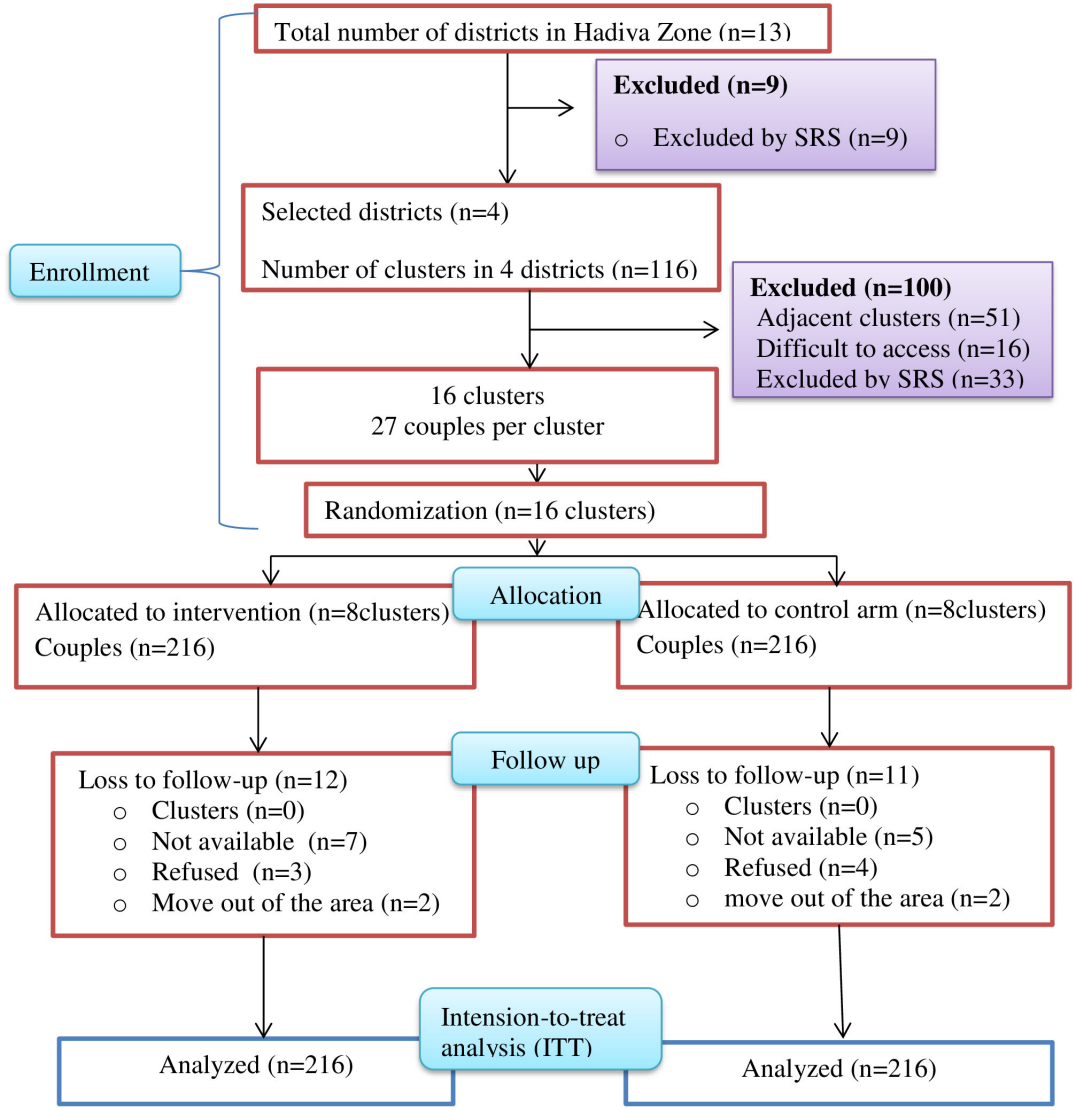
Consolidated standards of reporting trials flow diagram of participant recruitment, randomization, and cluster allocation in rural districts of Hadiya Zone, Central Ethiopia, 2023/2024.

The randomization process involved creating four blocks within each district, each containing four clusters, resulting in a total of 16 clusters. A statistician, blinded to the study groups, divided the four clusters in each stratum into blocks of size 2. From the two possible permutations within each block, the statistician determined the randomized sequence of clusters using sealed lots. Based on the selected permutation for each stratum, clusters within each block were allocated to either the intervention or control arms. This process was repeated for each stratum, resulting in the selection of two clusters from each district, totaling 8 clusters for the intervention arm and 8 clusters for the control arm, with a 1:1 allocation ratio. Neither the HEWs (interventionists) nor the data collectors were informed about the intervention hypothesis. The overall recruitment, randomization, and allocation of clusters are illustrated in the Consolidated Standards of Reporting Trials (CONSORT) flow diagram (Figure 2).

### Intervention description

In this study, CBVPE was implemented as an intervention for the intervention group. The intervention package was developed by reviewing various studies (44, 46). The intervention package consists of six educational topics: (i) overview of gender-based violence, violence against women, and IPV; (ii) common triggers of IPV and consequences; (iii) inequitable gender norms related to IPV; (iv) understanding of unhealthy and healthy marital relationship dynamics; (v) power and control in relationships and the importance of joint decision-making and its application; and (vi) effective communication and conflict resolution. Each educational topic had session objectives and a set of preventive messages.

The intervention was provided for couples (pregnant women and their male partners) for six consecutive months. All six education sessions were conducted from 13 through 36 weeks of gestational age. A group health education was provided to HEWs in a health post found in each selected cluster. An average of 60 min was spent for each session. Brainstorming, interactive lectures, questions and answers, role play, take-home exercises, and reflections were an integral part of all educational sessions. Flip charts and parkers were used during sessions. Posters that consist of an image and key message of the session were posted on the walls during each educational session and distributed to the participants. Neither the interventionists nor the outcome assessors were informed about the intervention hypothesis. The attendance sheet was used to assess the participants' adherence to the intervention package (Table 1).

**Table 1:** The intervention description summary for the effectiveness of couple-based violence prevention education on male partner knowledge, attitude, and controlling behavior, rural districts of Hadiya Zone, Central Ethiopia, 2023/2024.

**Content of intervention**	**Dosage**	**Frequency**	**Duration**	**Compliance results**
Couples-based health education + distribution of posters with key messages	55–60 min	6	6 month	1. 97.2% of women and 96.0% of men attended the education session 2. 100% of the participants received the posters

### Usual standard of care

In this study, the control group did not receive any component outline in the intervention package (CBVPE). Participants in this group received the usual standard of care provided by rural HEWs. The usual standard of care included preventive and promotional services, which are categorized under four main topic areas: family health services, disease prevention and control, hygiene and environmental sanitation, health education, and communication (50).

### Intervention fidelity

To maintain intervention fidelity, several measures were implemented. First, a clear trainer manual was developed to guide the interventionists. Additionally, the interventionists underwent extensive training provided by the principal investigator. Pre- and post-tests were administered to evaluate the interventionists' knowledge, and those who scored 75% and above were deemed eligible to deliver the intervention (51). During the sessions, on-site checks were conducted to ensure adherence, and feedback meetings with the interventionists were held at the end of each session. Furthermore, a fidelity checklist was used at the end of each session to assess the adherence and intensity of the intervention (52).

### Instrument translation, validity, and reliability

Translation of the questionnaire from the original English version into the local language is an important step in ensuring the instrument's validity (53). The methodological approach outlined by Beaton et al. (54) was adhered to in order to maintain conceptual, experiential, and idiomatic equivalence. The questionnaire was translated from English into the local language, Hadiyisa, by bilingual translators. Subsequently, it was translated back into English by bilingual translators from the Department of Hadiyisa at Wachemo University in Ethiopia. These translators were also blind to the original version. Back-translation is essential to achieve semantic equivalence. An expert committee meeting was convened to consolidate all language versions and finalize the pre-test questionnaire for field testing (55).

The original and back-translated English versions of the instruments were compared to ascertain the translation's accuracy. To validate the translated instrument, a method developed by Skperber et al. (56) was used to assess each item in the original and back-translated versions, ranking them based on language comparability and interpretive similarity. Experts fluent in English from the Department of Foreign Languages and Literatures evaluated the comparisons using Likert scales ranging from 1 (highly comparable/extremely similar) to 7 (not at all comparable/not at all similar). A formal review of the translation was conducted for any item with a mean score above 3.

The translated instrument was pilot-tested among 27 eligible couples (27 male partners and their wives) who were excluded from the main study to evaluate its clarity, comprehension, and appropriateness. Subject experts reviewed the content, and necessary revisions were made. Internal consistency was assessed using Cronbach's alpha, with a value of ≥ 0.7 indicating the instrument was reliable (57). The finalized questionnaires were then administered at the study's baseline and endline assessments.

### Timeline of the study

The study was conducted over a 10-month period. The participants' enrollment, intervention implementation, and assessment schedule were summarized in Table 2.

**Table 2:** Participants' timeline of enrolment, intervention, and assessment schedule in rural districts of Hadiya Zone, Central Ethiopia, 2023/2024.

**Activity**		**Study period**								
		**Enrolment**	**Allocation**	**Intervention**						**Close-out**
		**t**_(−1)_	**t**_(−2)_	**t**_1_	**t**_2_	**t**_3_	**t**_4_	**t**_5_	**t**_6_	**t** **_+2_**
Enrolment	Eligibility screen									
	Informed consent									
Allocation										
Baseline assessments									
Intervention										
Endline assessments										

The colors highlight different study activities at each time point. For example, yellow indicates enrolment-related tasks, light green represents intervention implementation, Orange represents allocation, and Blue marks assessment periods.

### Outcomes of interest and measurement

The outcomes of the study were knowledge of IPV, attitudes toward wife beating, and controlling behavior.

Knowledge of IPV was measured by asking the male partners about nine items of IPV knowledge questions. Respondents scoring approximately 50% and above of the correct answers were considered to have “good knowledge,” while those scoring <50% were classified as having “poor knowledge” (58).

Attitude toward IPV (wife beating) was measured by asking the male partners whether or not a husband is justified in hitting or beating his spouse in five scenarios: (a) “If she is going out without telling him?” (b) “If the wife neglects the children?” (c) “If the wife argues with the husband?” (d) “If the wife refuses to have sex with the husband?” (e) “If the wife burns the food?” Responses of “supportive attitudes toward IPV” to one or more of the scenarios were coded as 1; “not supportive attitudes toward IPV” to all scenarios were coded as 0 (59).

Controlling behavior was assessed by asking the male partners whether the husband is justified in controlling the wife in the following situations: (i) being jealous if she talked with other men, (ii) accusing her of unfaithfulness, (iii) not permitting her to meet her friends, (iv) trying to limit her contact with family, (v) insisting on knowing her whereabouts, and (vi) not trusting her with money. If the participant responded “yes” to one or more of the control questions, it was coded as 1. Otherwise, it was coded as “no” (0) (42).

### Data collection

Pre-tested, structured interviewer-administered questionnaires were used to collect data. The tool was adapted from the WHO multicounty study on women's health and domestic violence against women (60). Diploma-holder female nurses were used to collect data. The principal investigator provided two days of training to data collectors and supervisors. Baseline data were collected immediately after recruitment of the participants, while endline data was collected 2 months after completion of the intervention. Data was collected using the mobile-based application, KoBo Collect (61). Data was collected through home visits. Data were collected on various variables, such as sociodemographic and economic characteristics, lifestyle, knowledge, attitudes, and controlling behavior. Baseline and endline assessments were monitored and audited by field supervisors. Unique codes were assigned to participants and clusters for a complete questionnaire and securely stored on a password-protected data server, specifically the KoBo Toolbox to maintain confidentiality.

### Statistical analysis

The data analysis was performed using SPSS version 25. Descriptive statistics, such as proportions, percentages, means, and standard deviations, were computed to describe the characteristics of the participants in the intervention and control groups. The proportion of study participants who had good knowledge of IPV, supportive attitudes toward wife beating, and controlling behavior were computed at baseline and endline for the intervention and control groups. The Pearson's chi-square test was used to compare the characteristics of participants, including the outcomes of interest, in the intervention and control groups for categorical variables. Meanwhile, the independent sample *t*-test was used for continuous variables. The McNemar test was used to compare the outcomes of interest between the intervention and control groups before and after the intervention.

Data analysis was performed using an intention-to-treat (ITT) analysis approach. Multiple imputation techniques were applied to the handle missing data. A difference-in-difference analysis was conducted to determine the net effect of the intervention. We used a Generalized Estimating Equation (GEE) model to compare the odds of the outcomes between the intervention and control groups. In order to account for intra-cluster correlation, we used the autoregressive (AR-1) working correlation structure. We assessed the fit of the model using the value of Quasi-Likelihood under the Independence Model Criterion (QIC). Additionally, we performed sensitivity analyses to investigate the impact of missing data and different working correlation structures on the outcome of interest.

### Ethical approval

This study was approved by the Ethics Committee of the Institutional Review Board at Jimma University on November 8, 2022 (JUIH/IRB/222/2022). The protocol of the trial was registered on ClinicalTrials.gov as NCT 05856214 on May 4, 2023. The data collectors provided a clear explanation of the study's purpose and nature, emphasizing the participant's right to decline participation and choose not to answer certain questions as described in the information sheet. Participants were assured of the strict confidentiality of the information they provided. Data collection was conducted in a private setting to ensure their privacy and minimize the risk of disclosure. Written informed consent was obtained from participants prior to the interview. The study result will be disseminated to the study participants through community meetings organized by HEWs residing in the study clusters. The results were reported according to the CONSORT checklist for cluster randomized trials (62) (Supplementary Table S1).

## Result

### Sociodemographic, economic, and lifestyle characteristics of participants

The study enrolled a total of 432 couples, consisting of 432 male partners/husbands and 432 pregnant women, assigning 216 couples to the intervention group and 216 to the control group. At the endline, 94.4% of male partners in the intervention group and 94.9% of those in the control group were available for the intention-to-treat analysis. The study participants were all married men, aged between 15 and 55 years. In the intervention group, 56.9% of participants were between 25 and 34 years old, while 52.0% in the control group were within this age range. The mean age and standard deviation were 30.8 ± 6.2 years for the intervention group and 32.0 ± 6.8 years for the control group, with a *p*-value of 0.06. The majority of participants in both groups (60.2%) were Protestant Christians, with no statistically significant difference in religious affiliation between the intervention and control groups (*p* = 0.31). Additionally, there were no statistically significant differences between the intervention and control groups concerning other sociodemographic, economic, and lifestyle characteristics, including education, occupation, autonomy, socioeconomic status, cigarette smoking, and alcohol consumption, except for ethnicity at baseline (*p* = 0.02) (Table 3).

**Table 3:** Sociodemographic, economic, and lifestyle characteristics of the respondents at baseline, Hadiya Zone, Central Ethiopia, 2023/2024.

**Participants' characteristics**	**Categories**	**Intervention group (*n* = 216)*n* (%)**	**Control group (*n* =216)*n* (%)**	***P-*value**
Age group	15–24	30 (13.9%)	24 (11.1%)	0.20
	25–34	123 (56.9%)	112 (51.9%)	
	35–49	63 (29.2%)	80 (37.0%)	
	Mean ± SD	30.8 ± 6.2	32.0 ± 6.8	0.06
Age at marriage	<20	34(15.7%)	29 (13.4%)	0.49
	≥20	182 (84.3%)	187 (86.6%)	
	Mean ± SD	21.9 ± 3.0	22.3 ± 2.9	0.16
Ethnicity	Hadiya	168 (77.8)	156 (72.2)	0.02
	Kembata	19 (8.8)	38 (17.6)	
	Silte	14 (6.5)	14 (6.5)	
	Others^b^	15 (6.9)	8 (3.7)	
Religion	Protestant Christian	130 (60.2)	130 (60.2)	0.31
	Orthodox Christian	41 (19.0)	52 (24.1)	
	Others^a^	45 (20.8)	34 (15.7)	
Education	No education	81 (37.5)	76 (35.2)	0.51
	Elementary school	72 (33.3)	65 (30.1)	
	Junior or high school	39 (18.1)	41 (19.0)	
	College/higher	24 (11.1)	34 (15.7)	
Occupation	Farmer	127 (58.8)	126 (58.3)	0.67
	Merchant	61 (28.2)	56 (25.9)	
	Employed	28 (13.0)	34 (15.7)	
No of children alive	0–1	38 (17.6)	28 (13.0)	0.40
	2–4	90 (41.7)	97 (44.9)	
	5+	88 (40.7)	90 (42.1)	
	Mean ± SD	4.0 ± 2.1	4.2 ± 2.0	0.29
Household wealth index	Low	77 (35.5)	67 (31.0)	*0.59*
	Medium	70 (32.4)	74 (34.3)	
	High	69 (31.9)	75 (34.7)	
Marriage type	Monogamy	185 (85.6)	173 (82.9)	*0.12*
	Polygamy	31 (14.4)	43 (17.1)	
Smoking cigarrette	Yes	44 (20.4)	37 (17.1)	*0.34*
	No	172 (79.6)	179 (82.9)	
Alcohol consumption	Yes	59 (27.3)	58 (26.9)	*0.91*
	No	157 (73.7)	158 (73.1)	

a Amhara, Gurage, Oromo, Wolayita.

b Muslim, Catholic, Adventist, Apostolic; SD, Standard Deviation.

Pearson chi-square test, significant at *P* value <0.05.

### Male partners' knowledge and attitudes toward intimate partner violence and controlling behavior at baseline

At baseline, a significant proportion of participants (42.6% of male partners in the intervention group and 41.2% in the control group) had poor knowledge of IPV, and the difference between the two groups was not statistically significant (*p* = 0.77). The majority of respondents −87.5% of husbands in the intervention group and 89.4% in the control group—had heard of what IPV is (*p* = 0.55). However, less than half of the husbands in both groups (46.3% in the intervention group and 48.1% in the control group) were unaware that IPV is a violation of human rights (*p* = 0.70).

About 67.0% of male partners in the intervention group and 64.0% in the control group had a supportive attitude toward wife beating in at least one specific condition (*p* = 0.48). The most commonly mentioned justifications for wife-beating were “neglecting the children” (38.4% of male partners in the intervention group and 38.9% in the control group) and “going out without informing the husband” (27.3% in the intervention group and 29.6% in the control group).

Furthermore, at baseline, 59.0% of male partners in the intervention group and around 57.0% in the control group reported engaging in at least one controlling behavior. There was no statistically significant difference between the two groups (*p* = 0.62). The most frequently reported controlling behavior was being “jealous if the wife talked to other men,” with 39.4% in the intervention group and 43.1% in the control group. Again, there was no statistically significant difference between the two groups (*p* = 0.43) (Table 4).

**Table 4:** Knowledge and attitudes toward intimate partner violence and controlling behavior at baseline, Hadiya Zone, Central Ethiopia, 2023/2024.

**Variables**	**Categories**	**Intervention (*n* = 216)*n* (%)**	**Control (*n* = 216)*n* (%)**	***P*-value**
Knowledge	Good	124 (57.4)	127 (58.8)	0.77
	Poor	92 (42.6)	89 (41.2)	
Attitude	Supportive	145 (67.1)	138 (63.9)	0.48
	Not supportive	71 (32.9)	78 (36.1)	
Controlling behavior	Yes	128 (59.3)	123 (56.9)	0.62
	No	88 (40.7)	93 (43.1)	

### Intervention effectiveness

#### Male partners' knowledge, attitude toward intimate partner violence, and controlling behavior at the end line

At the endline, the knowledge of IPV among male partners significantly improved in the intervention group. In the intervention group, 84.7% of male partners had good knowledge, while 61.1% of male partners in the control group had good knowledge of IPV, with a statistically significant difference between the groups (*p* < 0.001). The intervention significantly reduced the male partners' attitudes toward wife beating. Eighty-three (38.4%) of participants in the intervention group had supportive attitudes toward wife beating in certain circumstances, while the attitude remained high (62.5%) in the control group (*p* < 0.001) (Table 5). Moreover, partners' controlling behavior was significantly reduced in the intervention group (37.5%) compared to the control group (55.6%), with a *p*-value of < 0.001.

**Table 5:** Male partners' knowledge, attitude, and controlling behavior at the end line, Hadiya Zone, Central Ethiopia, 2023/2024.

**Variables**	**Categories**	**Intervention (*n* = 216) *n* (%)**	**Control (*n* = 216)*n* (%)**	***P*-value**
Knowledge	Good	183 (84.7)	132 (61.1)	<0.001
	Poor	33 (15.3)	84 (38.9)	
Attitude	Supportive	83 (38.4)	135 (62.5)	<0.001
	Not supportive	133 (61.6)	81 (37.5)	
Controlling behavior	Yes	81 (37.5)	120 (55.6)	<0.001
	No	135 (62.5)	96 (44.4)	

The intervention group showed a significant improvement in participants' knowledge of IPV, increasing from 57.4% at baseline to 84.7% at the endline (*p* < 0.001). On the other hand, the control group only experienced a minimal improvement, with knowledge increasing from 58.8% at baseline to 61.1% at the endline, and this difference was not statistically significant. The intervention had a net effect of 25.0% in improving male partners' knowledge of IPV. Additionally, the intervention had a significant impact on male partners' attitudes toward wife beating, reducing from 67.1% at baseline to 38.4% at the endline (*p* < 0.001). In contrast, the control group showed no significant change, with attitudes changing from 63.9% at baseline to 62.5% at the endline (*p* = 0.69). The intervention had a net effect of reducing attitudes toward wife beating by 27.3%. Additionally, controlling behavior by male partners in the intervention group was significantly reduced from 59.3% at the beginning to 37.5% at the end (*p* < 0.001). In contrast, the control group showed a slight, insignificant reduction from 56.9% to 55.6% (*p* = 0.70). As a result, the intervention led to a net reduction of 20.5% in male partners controlling behavior (Table 6).

**Table 6:** The effect of couple-based violence prevention on male partners' knowledge, attitude, and controlling behavior, Hadiya Zone, Central Ethiopia, 2023/2024.

**Variables**	**Intervention group (n** = **216)**				**Control group (n** = **216)**				**DiD**
	**Base-line (*****n*** = **216)**	**End-line (*****n*** = **216)**	**Difference (EL-BL)**	***P*** **-value**	**Base- line (*****n*** = **216)**	**End-line (*****n*** = **216)**	**Difference (EL-BL)**	***P*** **-value**	
Knowledge	0.574	0.847	0.273	<0.001	0.588	0.611	0.023	0.30	0.25
Attitude	0.671	0.384	−0.287	<0.001	0.639	0.625	−0.014	0.69	−0.273
Controlling behavior	0.593	0.375	−0.218	<0.001	0.569	0.556	−0.013	0.70	−0.205

BL, Baseline; EL, Endline; Difference-in-Difference analysis, – (negative sign), decrease.

The analysis using the generalized estimating equation (GEE) model revealed that male partners in the intervention group were 3.7 times more likely to have good knowledge about IPV compared to male partners in the control group (AOR = 3.7; 95% CI 2.6–5.4). Moreover, male partners in the intervention group were 67.6% less likely to report a supportive attitude toward wife beating compared to male partners in the control group (AOR = 0.324; 95% CI 0.229–0.459). Also, the proportion of controlling behavior reported among male partners in the intervention group was 56.4% less compared to the control group (AOR = 0.436; 95% CI 0.317–0.600) (Table 7).

**Table 7:** Parameter estimates from a generalized estimating equation model demonstrating the effect of couple-based violence prevention education on male partners' knowledge and attitude toward intimate partner violence and controlling behavior, Central Ethiopia, 2023/2024.

**Variables**	**Arm**	**N**	**Adjusted odds ratio (AOR)**	**95%CI**	***P*-value**
Knowledge	Control	216	Ref		<0.001
	Intervention	216	3.7	2.6, 5.4	
Attitude	Control	216	Ref		<0.001
	Intervention	216	0.324	0.229, 0.459	
Controlling behavior	Control	216	Ref		<0.001
	Intervention	216	0.436	0.317, 0.600	

The model included time points, study group, and time points × study group; Ref, Reference category.

### Sensitivity analysis

We tested the GEE model using a variety of working correlation structures in our sensitivity analysis. The QIC values were constant across all tested correlation structures, and no variability in the parameter estimates was noted. Furthermore, we considered the presence of missing data in our sensitivity analysis. We observed that the parameter estimates remained unchanged whether the missing data were included (imputed cases) or excluded (complete cases) from the model analysis.

## Discussion

The trial revealed that the CBVPE was successful in improving the male partners' knowledge of IPV. Additionally, the intervention was effective in reducing the male partners' attitudes toward wife beating and their controlling behavior. At baseline, approximately 57.0% of the participants in the intervention group and about 59.0% of the participants in the control group had good knowledge of IPV. Moreover, a sizable percentage of male partners (67.1% and 59.3% in the intervention group, and 63.9% and 56.9% in the control group) endorsed wife beating and controlling behavior for at least one reason. However, the intervention group showed significant improvement in the male partners' knowledge and in reducing attitudes toward wife beating and controlling behavior.

The male partners' knowledge about IPV was significantly improved in the intervention group compared to those in the control group. The net increment of the knowledge of IPV due to the intervention was 25.0%. Moreover, male partners in the intervention group were nearly four times more likely to have good knowledge about IPV compared to those in the control group. This implies the significance of targeted educational interventions in addressing IPV and suggests that engaging men in prevention efforts can result in meaningful enhancements in knowledge about IPV and potentially lead to a decrease in IPV against women (43). Hence, creating awareness programs through engaging men can help them to have a better understanding of IPV, its forms, causes, and consequences.

Moreover, in this study, male partners' supportive attitudes toward wife beating were significantly reduced in the intervention group as compared to the control group. The net effect of the intervention in reducing male partners' supportive attitudes toward wife beating at certain circumstances was about 27.0%. Also, male partners in the intervention group were 67.6% less likely to report supportive attitudes toward wife beating compared to those in the control group. This underscores that giving health education on violence prevention changes the male partners' attitudes toward wife beating. In observational studies, attitudes toward wife-beating have consistently been identified as a key predictor of the prevalence of IPV (28, 29, 39, 40). Therefore, providing couple-based violence prevention education can play a vital role in reshaping male partners' attitudes toward IPV and potentially reducing its occurrence (41, 63).

A sizable percentage of male partners-−59.3% in the intervention group and about 57.0% in the control group—endorsed the controlling behavior for at least one condition. The most frequently cited justification for controlling wives in both the intervention group (39.4%) and the control group (43.1%) was “jealousy or anger if the wives talk with other men”. This finding is consistent with the Ethiopian Demographic and Health Survey (2016), where 39.0% of participants similarly reported that jealousy arising from wives talking with other men was a precondition for husbands controlling (33). However, the male partners controlling behavior significantly reduced in the intervention group compared to those in the control group. The net reduction in controlling behavior due to the intervention was 20.5%. In addition, male partners in the intervention group were 56.4% less likely to report controlling behavior compared to male partners in the intervention group. This finding suggests that educating couples on violence prevention can effectively decrease controlling behaviors in male partners. Controlling behaviors are known to be significant factors contributing to intimate partner violence (30, 34, 42, 64). Therefore, this education can play a crucial role in preventing such violence.

Our findings indicate that while a proportion of participants approve of IPV, approval alone does not fully explain IPV perpetration or victimization. Beyond the attitudinal acceptance and controlling behavior, studies also highlight that economic dependence, substance use; gender norms, poor communication skills, power imbalance, and lack of support systems contribute to both the perpetration and experience of IPV. These structural and behavioral factors interact in complex ways, reinforcing cycles of violence. Our study intervention addressing the male partners' knowledge, attitude, and controlling behavior may not be sufficient in preventing or reducing IPV. Programs such as counseling interventions (65), women empowerment (66), couples-focused training (46), and community mobilization and group-based interventions have shown promise in reducing IPV (67).

This study had strengths. We employed a gold standard design—a cluster-randomized controlled trial. This design was used to properly determine the intervention effect and account for any confounders, ensuring the validity of the results. The study was carried out in compliance with the published study protocol (49). Nevertheless, this study has a few limitations. Due to the nature of the study, we were unable to blindly assign the intervention to the participants, although the intervention hypothesis was not disclosed to the interventionists or outcome assessors. Additionally, there was no evaluation of attitudes regarding gender equity and variables associated with socio-emotional skills that have also been shown to intervene in IPV were not evaluated. Finally, the sensitive nature of the topic may have introduced social desirability bias.

## Conclusion

The intervention proved effective in enhancing knowledge about IPV, reducing supportive attitudes toward wife beating, and curbing controlling behaviors among male partners. This approach holds promise for scaling up and adapting to similar contexts in Ethiopia, offering a viable strategy to address male partners' knowledge, attitudes, and controlling behaviors.

## Data Availability

The raw data supporting the conclusions of this article will be made available by the authors, without undue reservation.

## References

[1] Lelaurain S Graziani P Lo Monaco G. Intimate partner violence and help-seeking. Eur Psychol. (2017) 22:263–81. 10.1027/1016-9040/a000304

[2] WHO. Violence Against Women Prevalence Estimates, 2018: Global, Regional and National Prevalence Estimates for Intimate Partner Violence Against Women and Global and Regional Prevalence Estimates for Non-Partner Sexual Violence Against Women. Executive Summary. Geneva: World Health Organization (2021). Available at: https://www.who.int/publications/i/item/9789240022256 (acccessesd October 7, 2022).

[3] McQuigg RJA. Domestic violence as a human rights issue: rumor v. Italy. Eur J Int Law. (2016) 26:1009–25. 10.1093/ejil/chv057

[4] Guedes A Bott S Garcia-Moreno C Colombini M. Bridging the gaps: a global review of intersections of violence against women and violence against children. Glob Health Action. (2016) 9:31516. 10.3402/gha.v9.31516 27329936 PMC4916258

[5] Campbell JC. Health consequences of intimate partner violence. Lancet. (2002) 359:1331–6. 10.1016/S0140-6736(02)08336-8 11965295

[6] Stockman JK Hayashi H Campbell JC. Intimate partner violence and its health impact on ethnic minority women [corrected]. J Womens Health. (2015) 24:62–79. 10.1089/jwh.2014.4879 25551432 PMC4302952

[7] Stubbs A Szoeke C. The effect of intimate partner violence on the physical health and health-related behaviors of women: a systematic review of the literature. Trauma Violence Abuse. (2022) 23:1157–72. 10.1177/1524838020985541 33541243

[8] Fan S Koski A. The health consequences of child marriage: a systematic review of the evidence. BMC Public Health. (2022) 22:309. 10.1186/s12889-022-12707-x 35164724 PMC8845223

[9] Martin SL Beaumont JL Kupper LL. Substance use before and during pregnancy: links to intimate partner violence. Am J Drug Alcohol Abuse. (2003) 29:599–617. 10.1081/ADA-120023461 14510043

[10] Wessells MG Kostelny K. The psychosocial impacts of intimate partner violence against women in LMIC contexts: toward a holistic approach. Int J Environ Res Public Health. (2022) 19:14488. 10.3390/ijerph192114488 36361364 PMC9653845

[11] Bonomi AE Anderson ML Rivara FP Thompson RS. Health care utilization and costs associated with physical and nonphysical-only intimate partner violence. Health Serv Res. (2009) 44:1052–67. 10.1111/j.1475-6773.2009.00955.x 19674432 PMC2699921

[12] Hisasue T Kruse M Hietamäki J Raitanen J Martikainen V Pirkola S. Health-related costs of intimate partner violence: using linked police and health registers. J Interpers Viol. (2024) 39:1596–622. 10.1177/08862605231211932 37978834

[13] Peterson C Kearns MC McIntosh WL Estefan LF Nicolaidis C McCollister KE. Lifetime economic burden of intimate partner violence among U. S. adults. Am J Prevent Med. (2018) 55:433–44. 10.1016/j.amepre.2018.04.049 30166082 PMC6161830

[14] Zegeye B Shibre G Ahinkorah BO Keetile M Yaya S. Urban-rural disparities in wife-beating attitude among married women: a decomposition analysis from the 2017 Senegal Continuous Demographic and Health Survey. Arch Public Health. (2021) 79:102. 10.1186/s13690-021-00612-5 34130759 PMC8204494

[15] Gundumogula M. Importance of focus groups in qualitative research. HE Int J Humanit Soc Stud. (2020) 8:82. 10.24940/theijhss/2020/v8/i11/HS2011-082

[16] Kebede AA Aklil MB Gessesse DN Tsega NT Temesgan WZ Abegaz MY. Nearly half of women have experienced intimate partner violence during pregnancy in Northwest Ethiopia, 2021; the role of social support and decision-making power. Front Public Health. (2022) 10:904792. 10.3389/fpubh.2022.904792 35844863 PMC9280332

[17] Liyew AM Alem AZ Ayalew HG. Magnitude and factors associated with intimate partner violence against pregnant women in Ethiopia: a multilevel analysis of 2016 Ethiopian demographic and health survey. BMC Public Health. (2022) 22:284. 10.1186/s12889-022-12720-0 35148725 PMC8840032

[18] López-Goñi JJ Musa A Chojenta C Loxton D. High rate of partner violence during pregnancy in eastern Ethiopia: findings from a facility-based study. PLoS ONE. (2020) 15:e0233907. 10.1371/journal.pone.0233907 32497059 PMC7272015

[19] Lencha B Ameya G Baresa G Minda Z Ganfure G. Intimate partner violence and its associated factors among pregnant women in Bale Zone, Southeast Ethiopia: a cross-sectional study. PLoS ONE. (2019) 14:e0214962. 10.1371/journal.pone.0214962 31042713 PMC6494036

[20] Gebreslasie KZ Weldemariam S Gebre G Zenebe D Mehari M Birhane A. Intimate partner violence during pregnancy and risks of low birth weight and preterm birth in hospitals of Tigray, Northern Ethiopia. Sci Rep. (2024) 14:1363. 10.1038/s41598-024-51569-8 38228730 PMC10791596

[21] Alebel A Kibret GD Wagnew F Tesema C Ferede A Petrucka P. Intimate partner violence and associated factors among pregnant women in Ethiopia: a systematic review and meta-analysis. Reprod Health. (2018) 15:196. 10.1186/s12978-018-0637-x 30514311 PMC6278116

[22] Adhena G Oljira L Dessie Y Hidru HD. Magnitude of intimate partner violence and associated factors among pregnant women in Ethiopia. Adv Public Health. (2020) 2020:1–9. 10.1155/2020/1682847

[23] Diddy Antai Adaji S. Community-level influences on women's experience of intimate partner violence and terminated pregnancy in Nigeria: a multilevel analysis. BMC Preg Childb. (2012) 12:128. 10.1186/1471-2393-12-128 23150987 PMC3541204

[24] Alhusen JL Ray E Sharps P Bullock L. Intimate partner violence during pregnancy: maternal and neonatal outcomes. J Womens Health. (2015) 24:100–6. 10.1089/jwh.2014.4872 25265285 PMC4361157

[25] Agde ZD Magnus JH Assefa N Wordofa MA. Community perspectives on intimate partner violence during pregnancy: a qualitative study from rural Ethiopia. Int J Environm Res Public Health. (2025) 22:197. 10.3390/ijerph22020197 40003422 PMC11855346

[26] Farrington DP Gaffney H Ttofi MM. Systematic reviews of explanatory risk factors for violence, offending, and delinquency. Aggress Violent Behav. (2017) 33:24–36. 10.1016/j.avb.2016.11.004

[27] Garzón Segura AM Carcedo González RJ. Effectiveness of a prevention program for gender-based intimate partner violence at a colombian primary school. Front Psychol. (2019) 10:3012. 10.3389/fpsyg.2019.03012 32038389 PMC6985582

[28] Tanya Abramsky CHW Garcia-Moreno C Devries K Kiss L Ellsberg M Jansen H. What factors are associated with recent intimate partner violence? Findings from the WHO multi-country study on women's health and domestic violence. BMC Public Health. 11:109. 10.1186/1471-2458-11-109 21324186 PMC3049145

[29] Pradhan MR De P. Men's attitude towards wife-beating: understanding the pattern and trend in India. BMC Public Health. (2024) 24:331. 10.1186/s12889-024-17782-w 38297338 PMC10829205

[30] Baskan B Alkan O. Determinants of intimate partner controlling behavior targeting women in Turkiye. Front Psychol. (2023) 14:1174143. 10.3389/fpsyg.2023.1174143 37284474 PMC10239945

[31] Shakya HB Cislaghi B Fleming P Levtov RG Boyce SC Raj A. Associations of attitudes and social norms with experiences of intimate partner violence among married adolescents and their husbands in rural Niger: a dyadic cross-sectional study. BMC Womens Health. (2022) 22:180. 10.1186/s12905-022-01724-y 35585589 PMC9118706

[32] Perrin N Marsh M Clough A Desgroppes A Yope Phanuel C Abdi A. Social norms and beliefs about gender based violence scale: a measure for use with gender based violence prevention programs in low-resource and humanitarian settings. Confl Health. (2019) 13:6. 10.1186/s13031-019-0189-x 30899324 PMC6408811

[33] CSA. Ethiopia Demographic and Health Survey 2016. Addis Ababa, Ethiopia, and Rockville, Maryland, USA: CSA and ICF. 2016. Available at https://dhsprogram.com/pubs/pdf/fr328/fr328.pdf (accessed September 12, 2022).

[34] Mukherjee R Joshi RK. Controlling behavior and intimate partner violence: a cross-sectional study in an urban area of Delhi, India. J Interpers Violence. (2021) 36:Np10831–np42. 10.1177/0886260519876720 31561731

[35] Machado DF Castanheira ERL Almeida MAS. Intersections between gender socialization and violence against women by the intimate partner. Cien Saude Colet. (2021) 26:5003–12. 34787193 10.1590/1413-812320212611.3.02472020

[36] Connell RW. Masculinities. Berkeley Los Angeles: University of California Press. (1995). Available at: https://www.ucpress.edu/books/masculinities/paper (accessed February 19, 2024).

[37] Jewkes R Sikweyiya Y Morrell R Dunkle K. Gender inequitable masculinity and sexual entitlement in rape perpetration South Africa: findings of a cross-sectional study. PLoS ONE. (2011) 6:e29590. 10.1371/journal.pone.0029590 22216324 PMC3247272

[38] Fleming PJ Lee JG Dworkin SL. “Real men don't”: constructions of masculinity and inadvertent harm in public health interventions. Am J Public Health. (2014) 104:1029–35. 10.2105/AJPH.2013.301820 24825202 PMC4062033

[39] Bengesai AV Khan HTA. Exploring the association between attitudes towards wife beating and intimate partner violence using a dyadic approach in three sub-Saharan African countries. BMJ Open. (2023) 13:e062977. 10.1136/bmjopen-2022-062977 37316321 PMC10277069

[40] Tsawe M Mhele K. Determinants of wife-beating justification amongst men in southern African countries: Evidence from demographic and health surveys. Afr J Reprod Health. (2022) 26:85–93. 10.29063/ajrh2022/v26i9.9 37585073

[41] Kadengye DT Izudi J Kemigisha E Kiwuwa-Muyingo S. Effect of justification of wife-beating on experiences of intimate partner violence among men and women in Uganda: A propensity-score matched analysis of the 2016 Demographic Health Survey data. PLoS ONE. (2023) 18:e0276025. 10.1371/journal.pone.0276025 37043482 PMC10096235

[42] Antai D. Controlling behavior, power relations within intimate relationships and intimate partner physical and sexual violence against women in Nigeria. BMC Public Health. (2011) 11:511. 10.1186/1471-2458-11-511 21714854 PMC3161889

[43] Reyal HP Perera MN Guruge GND. Effectiveness of a community-based participatory health promotion intervention to address knowledge, attitudes and practices related to intimate partner violence: a quasi-experimental study. BMC Public Health. (2024) 24:1417. 10.1186/s12889-024-18893-0 38802834 PMC11131198

[44] Sharma V Leight J Verani F Tewolde S Deyessa N. Effectiveness of a culturally appropriate intervention to prevent intimate partner violence and HIV transmission among men, women, and couples in rural Ethiopia: Findings from a cluster-randomized controlled trial. PLoS Med. (2020) 17:e1003274. 10.1371/journal.pmed.1003274 32810146 PMC7433859

[45] Casey E Carlson J Two Bulls S Yager A. Gender transformative approaches to engaging men in gender-based violence prevention: a review and conceptual model. Trauma Viol Abuse. (2018) 19:231–46. 10.1177/1524838016650191 27197533

[46] Dunkle K Stern E Chatterji S Heise L. Effective prevention of intimate partner violence through couples training: a randomised controlled trial of Indashyikirwa in Rwanda. BMJ Glob Health. (2020) 5:2439. 10.1136/bmjgh-2020-002439 33355268 PMC7757483

[47] Karakurt G Whiting K van Esch C Bolen SD Calabrese JR. Couples therapy for intimate partner violence: a systematic review and meta-analysis. J Marital Fam Ther. (2016) 42:567–83. 10.1111/jmft.12178 27377617 PMC5050084

[48] UNDP. Sustainable Development Goals (SDGs). (2015). Available at: https://sustainabledevelopment.un.org/content/documents/1684SF_-SDG_Universality_Report_-_May_2015.pdf (accessed October 26, 2022).

[49] Agde ZD Magnus JH Assefa N Wordofa MA. The protocol for a cluster randomized controlled trial to evaluate couple-based violence prevention education and its ability to reduce intimate partner violence during pregnancy in Southwest Ethiopia. PLoS ONE. (2024) 19:e0303009. 10.1371/journal.pone.0303009 38739581 PMC11090299

[50] Basha SY Desta SH. The role of health extension workers in primary health care in Asgedetsi'mbla district: a case of lim'at t'abya health post. Int J Soc Sci Managem. (2017) 4:248–66. 10.3126/ijssm.v4i4.18504

[51] WHO. Evaluating Training in WHO. (2010). Available at: https://iris.who.int/bitstream/handle/10665/70552/WHO_HSE_GIP_ITP_2011.2_eng.pdf (accessed July 9, 2022).

[52] Breitenstein SM Gross D Garvey CA Hill C Fogg L Resnick B. Implementation fidelity in community-based interventions. Res Nurs Health. (2010) 33:164–73. 10.1002/nur.20373 20198637 PMC3409469

[53] Wild D Grove A Martin M Eremenco S McElroy S Verjee-Lorenz A. Principles of good practice for the translation and cultural adaptation process for patient-reported outcomes (PRO) measures: report of the ISPOR task force for translation and cultural adaptation. Value Health. (2005) 8:94–104. 10.1111/j.1524-4733.2005.04054.x 15804318

[54] Beaton DEB Bombardier C Guillemin F Bosi Ferraz M. Guidelines for the process of cross-cultural adaptation of self-report measures. SPINE. (2000) 25:3186–91. 10.1097/00007632-200012150-00014 11124735

[55] Abdulahi M Fretheim A Magnus JH. Effect of breastfeeding education and support intervention (BFESI) versus routine care on timely initiation and exclusive breastfeeding in Southwest Ethiopia: study protocol for a cluster randomized controlled trial. BMC Pediatr. (2018) 18:313. 10.1186/s12887-018-1278-5 30257661 PMC6158863

[56] Sperber AD Devellis RF Boehlecke B. Cross-cultural translation. J Cross Cult Psychol. (2016) 25:501–24. 10.1177/0022022194254006

[57] Sharma B A. focus on reliability in developmental research through Cronbach's Alpha among medical, dental and paramedical professionals. Asian Pacific J Health Sci. (2016) 3:271–8. 10.21276/apjhs.2016.3.4.43

[58] Oche OM Adamu H Abubakar A Aliyu MS Dogondaji AS. Intimate partner violence in pregnancy: knowledge and experiences of pregnant women and controlling behavior of male partners in Sokoto, Northwest Nigeria. Int J Reprod Med. (2020) 2020:1–10. 10.1155/2020/7626741 32206671 PMC7079217

[59] Trott CD Harman JJ Kaufman MR. Women's attitudes toward intimate partner violence in Ethiopia: the role of social norms in the interview context. Viol Against Women. (2017) 23:1016–36. 10.1177/1077801216654018 27364004

[60] WHO. WHO Multi-country Study on Women's Health and Domestic Violence against Womeny. (2010). p. 65. Available at https://www.who.int/publications/i/item/9241593512 (accessed December 23, 2022).

[61] Olajide V. data_collection_KoboToolbox.pdf. (2019). Available at: https://www.researchgate.net/publication/335147345_Data_Collection_with_KoboToolbox (accessed July 10, 2022).

[62] Kenneth F Schulz DGA David Moher for the CONSORT Group. CONSORT 2010 Statement: updated guidelines for reporting parallel group randomised trials. BMC Med. 8:18. 10.1186/1741-7015-8-18 20334633 PMC2860339

[63] Noda M Ishida A. Changes in attitude toward intimate partner violence in rapidly developing countries: the case of Indonesia. Administ Sci. (2024) 14:100. 10.3390/admsci14050100

[64] Aizpurua E Copp J Ricarte JJ Vazquez D. Controlling behaviors and intimate partner violence among women in spain: an examination of individual, partner, and relationship risk factors for physical and psychological abuse. J Interpers Viol. (2021) 36:231–54. 10.1177/0886260517723744 29294888

[65] Arora S Deosthali PB Rege S. Effectiveness of a counselling intervention implemented in antenatal setting for pregnant women facing domestic violence: a pre-experimental study. BJOG Int J Obstet Gynaecol. (2019) 126 Suppl 4:50–7. 10.1111/1471-0528.15846 31257691

[66] Cripe SM Sanchez SE Sanchez E Ayala Quintanilla B Hernández Alarcon C Gelaye B. Intimate partner violence during pregnancy: a pilot intervention program in Lima, Peru. J Interpers Violence. (2010) 25:2054–76. 10.1177/0886260509354517 20145196 PMC3741342

[67] Leight J Cullen C Ranganathan M Yakubovich A. Effectiveness of community mobilisation and group-based interventions for preventing intimate partner violence against women in low- and middle-income countries: a systematic review and meta-analysis. J Glob Health. (2023) 13:04115. 10.7189/jogh.13.04115 37861113 PMC10588291

